# The effect of sagittal alignment, coronal balance, and segmental stability on preoperative patient-reported outcomes in patients with degenerative lumbar spondylolisthesis

**DOI:** 10.1186/s12893-023-01947-2

**Published:** 2023-03-07

**Authors:** Dong-Fan Wang, Xiao-Long Chen, Di Han, Chao Kong, Shi-Bao Lu

**Affiliations:** 1 Department of Orthopedics, Xuanwu Hospital, Capital Medical University, No.45 Changchun Street, Xicheng District, Beijing, 100053 China; 2 National Center for Clinical Research On Geriatric Diseases, No.45 Changchun Street, Xicheng District, Beijing, China

**Keywords:** Degenerative, Lumbar spondylolisthesis, Alignment, Patient-reported outcomes, Influencing factors

## Abstract

**Objective:**

The aim of this study was to investigate the association between spinal alignment and preoperative patient-reported outcomes (PROs) in patients with degenerative lumbar spondylolisthesis (DLS) and to identify the independent risk factors for worse preoperative PROs.

**Methods:**

In total, 101 patients suffering from DLS were retrospectively studied within a single medical center. Age, sex, height, weight, and body mass index were uniformly recorded. PRO-related indicators include the Oswestry Disability Index (ODI), the Japanese Orthopedic Association’s (JOA) score, and the visual analog scale (VAS) for back and leg pain. Sagittal alignment, coronal balance, and stability of the L4/5 level were evaluated through whole-spine anteroposterior and lateral radiographs and dynamic lumbar X-ray.

**Results:**

Increasing age (*P* = 0.005), higher sagittal vertical axis (SVA) (*P* < 0.001), and global coronal imbalance (GCI) (*P* = 0.023) were independent risk factors for higher ODI. Patients with GCI had lower JOA scores (*P* = 0.001) than those with balanced coronal alignment. Unstable spondylolisthesis (*P* < 0.001) and GCI (*P* = 0.009) were two vital predictors of VAS-back pain. Increasing age (*P* = 0.031), local coronal imbalance (LCI) (*P* < 0.001), and GCI (*P* < 0.001) were associated with higher VAS-leg pain. Moreover, patients with coronal imbalance also exhibited significant sagittal malalignment based on the subgroup analysis.

**Conclusion:**

DLS patients with higher SVA, unstable spondylolistheses, a combination of LCI/GCI, or increasing age were predisposed to have more severe subjective symptoms before surgery.

**Supplementary Information:**

The online version contains supplementary material available at 10.1186/s12893-023-01947-2.

## Introduction

Degenerative lumbar spondylolisthesis (DLS) is one of the most common pathologic conditions of the lumbar spine involving anterior displacement of a superior vertebra over the adjacent caudal vertebra with an intact neural arch [1, 2]. As reported by Rosenberg, the DLS occurs four-fold more frequently in females than males and up to nine times more frequently at the L4/5 level than in adjacent segments [3]. Disc degeneration was thought to be the initial event of DLS, and secondary changes, including osteophyte formation, ligament hypertrophy, and facet arthrosis, might appear gradually with the progression of degeneration [4]. Patients with DLS often complain of low back pain, neurogenic claudication, or radicular pain caused by abnormal stress distribution or nerve compression [5]. According to a widely accepted grading system proposed by Meyerding et al. [6], the severity of DLS is categorized into four degrees, and the vast majority of patients are degree I with a slippage of less than 25% [7].

Currently, radiographic parameters are increasingly being utilized to evaluate DLS with the rapid development of radiological technology. Spinal sagittal alignment is one of the most vital aspects of imaging assessment [8–10]. In accordance with the classification system proposed by Gille et al., patients with DLS were divided into harmonious, compensatory, and imbalanced stages depending on the value of pelvic incidence (PI) minus lumbar lordosis (LL) mismatch and sagittal vertical axis (SVA), and the increase of preoperative Oswestry Disability Index (ODI) with the extent of sagittal malalignment was observed [11, 12]. In terms of inherent stability, another category scheme reported by Simmonds et al. suggested that patients who exhibit unstable spondylolisthesis are often accompanied by apparent preoperative low-back pain and have higher disease severity than those with a stable segment [13]. Furthermore, the combination of malalignment in the coronal plane in patients with DLS might also lead to worse ODI, visual analog scale (VAS) for back pain, and VAS-leg pain during the preoperative period, as presented by Pan et al. [14]. Given that the predominance or severity of symptoms might affect surgical efficacy [15, 16], surgeons should comprehensively evaluate the factors described above. However, a broad consensus has yet to be reached concerning the contributing indicators of the deterioration of preoperative clinical symptoms.

Consequently, we retrospectively reviewed the basic information and evaluated the radiographic data of patients with DLS who received treatment in our medical center. The present study was conducted to (1) investigate the correlation between spinal alignment and preoperative patient-reported outcomes (PROs) in patients with DLS and then (2) identify the independent influencing indicators for worse preoperative PROs.

## Materials and methods

### Patient population

After approval by the ethics committee in our hospital, a retrospective review of 247 consecutive patients who were diagnosed with DLS between January 2019 and December 2021 was performed. All patients were recruited from outpatient spinal surgery clinics and scheduled to undergo spine surgery. 3D printing molds are used for preoperative planning and surgical simulation.

The inclusion requirements included: (1) L4 DLS; (2) Meyerding grade I or II; and (3) complete radiographic data, including whole-spine anteroposterior and lateral radiographs and lateral flexion and extension X-ray of the lumbar spine.

The exclusion criteria were as follows: (1) multilevel DLS; (2) isthmic spondylolisthesis; (3) history of prior lumbar trauma, tumor, infection, cauda equina syndrome, or revision surgery; and (4) surgery requiring more than two-level instrumentation and fusion.

Demographic data were collected using electronic medical record reviews, including age, gender, height, weight, and body mass index. Overall, 101 patients were ultimately enrolled in the present study.

### Radiological data acquisition

#### Sagittal parameters

**Fig. 1:**
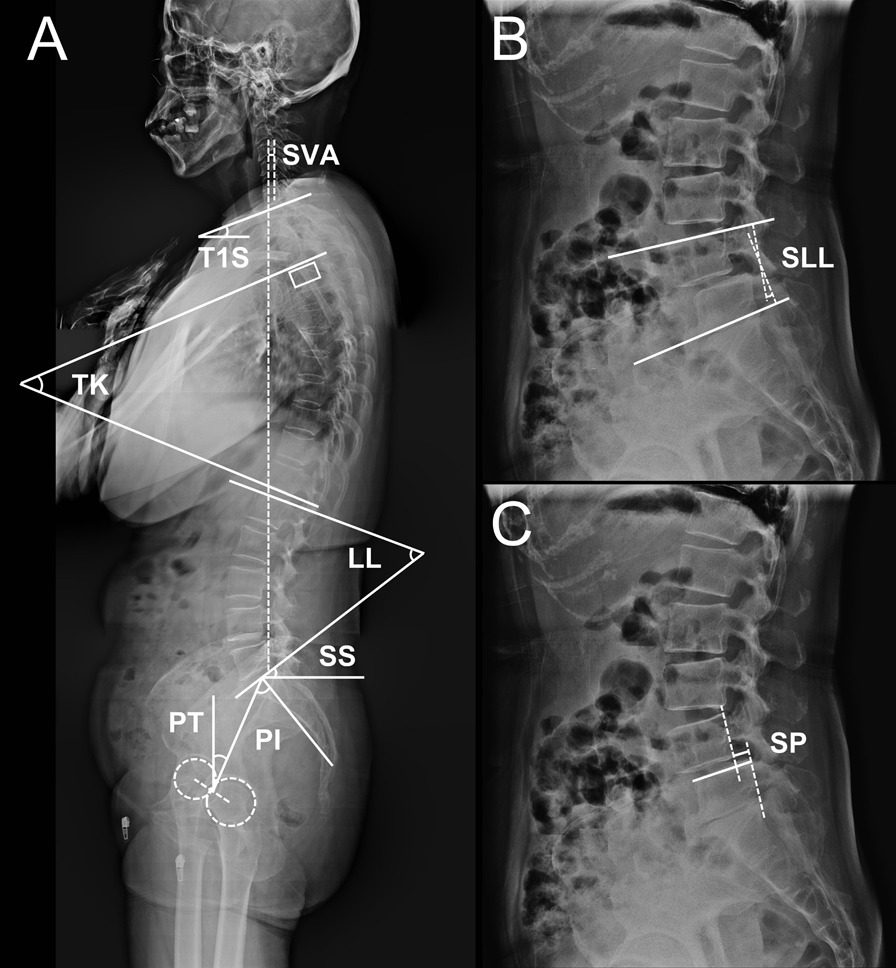
Measurements of sagittal parameters enrolled in the present study. **A** Global sagittal parameters. SVA, sagittal vertical axis; T1S, T1 slope; TK, thoracic kyphosis; LL, lumbar lordosis; SS, sacral slope; PT, pelvic tilt; PI, pelvic incidence. **B** SLL, segmental lumbar lordosis. **C** SP, slip percentage

Measurements of spinal sagittal parameters were illustrated in Fig. 1, covering: (1) T1 slope (T1S), the angle between the superior endplate of T1 and the horizontal line; (2) thoracic kyphosis (TK), the Cobb angle between the superior endplate of T4 and the inferior endplate of T12; (3) LL, the Cobb angle between the superior endplates of both L1 and S1; (4) sacral slope (SS), the angle between the superior endplate of the sacrum and the horizontal line; (5) pelvic tilt (PT), the angle between the line linking the midpoint of the superior endplate of S1 and the center of the femoral heads and vertical line; (6) PI, the angle between the line linking the midpoint of the superior endplate of S1 and the center of the femoral heads and the line vertical to the superior endplate of the sacrum; (7) SVA, the distance between the posterosuperior corner of S1 and the vertical line from the C7 body center; (8) segmental lumbar lordosis (SLL), the Cobb angle between the superior endplate of L4 and the inferior endplate of L5; and (9) slip percentage (SP), the ratio of the interval between two extended lines of the posterior aspect of L4 and L5 to the length of the superior endplate of L5. The ratio of PT to PI (PT/PI) suggests the degree of pelvic compensation [17].

#### Local and global coronal imbalance

**Fig. 2:**
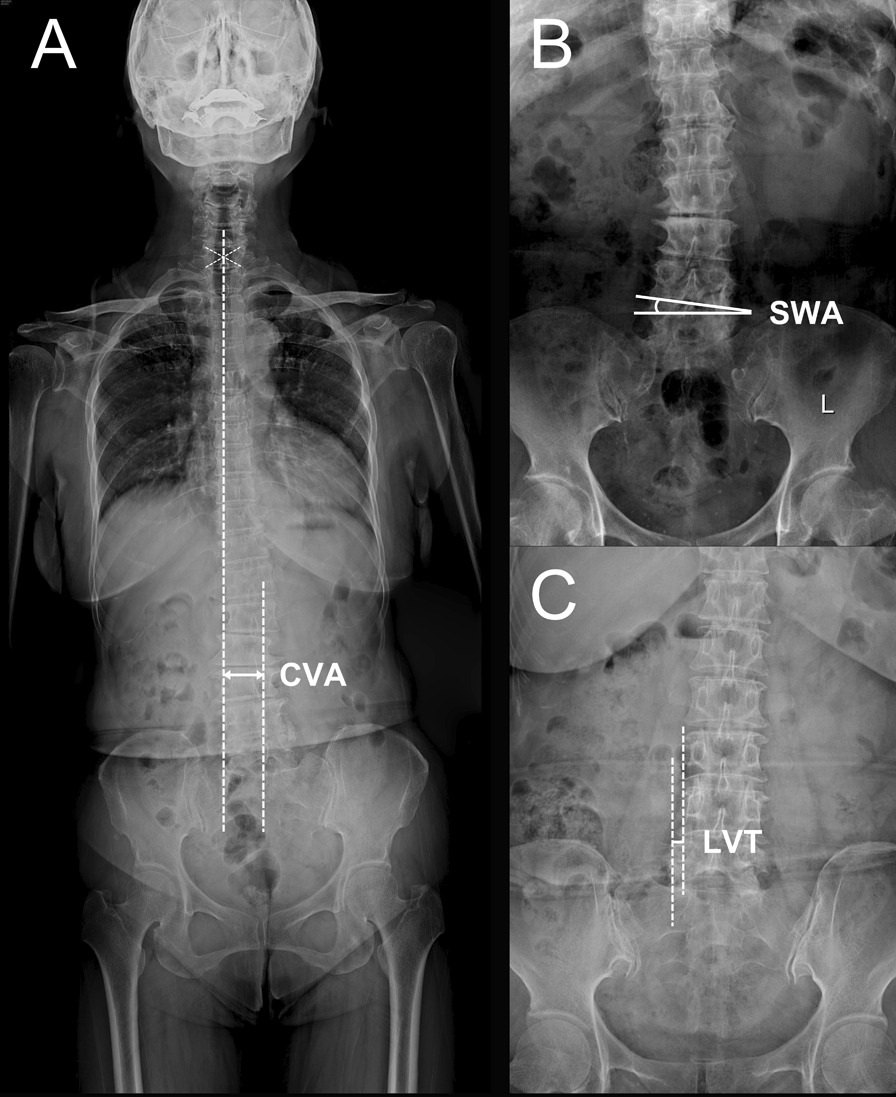
Measurements of coronal parameters enrolled in the present study. **A** CVA, coronal vertical axis; LCVA, lumbar coronal vertical axis. **B** SWA, segmental wedging angle. **C** LVT, lateral vertebral translation

Standing anteroposterior radiographs were used to measure the spinal coronal parameters (Fig. 2), including: (1) segmental wedging angle (SWA), the angle between the inferior endplate of L4 and the superior endplate of L5; (2) lateral vertebral translation (LVT), the distance between two extended lines of the lateral aspect of L4 and L5; and (3) coronal vertical axis (CVA), the distance between the center of S1 and the vertical line from the C7 body center. Local coronal imbalance (LCI) was defined as SWA > 5° or LVT > 5 mm, [14, 18] and global coronal imbalance (GCI) was defined as LCI combined with CVA > 30 mm [19].

#### Stable and unstable spondylolisthesis

**Fig. 3:**
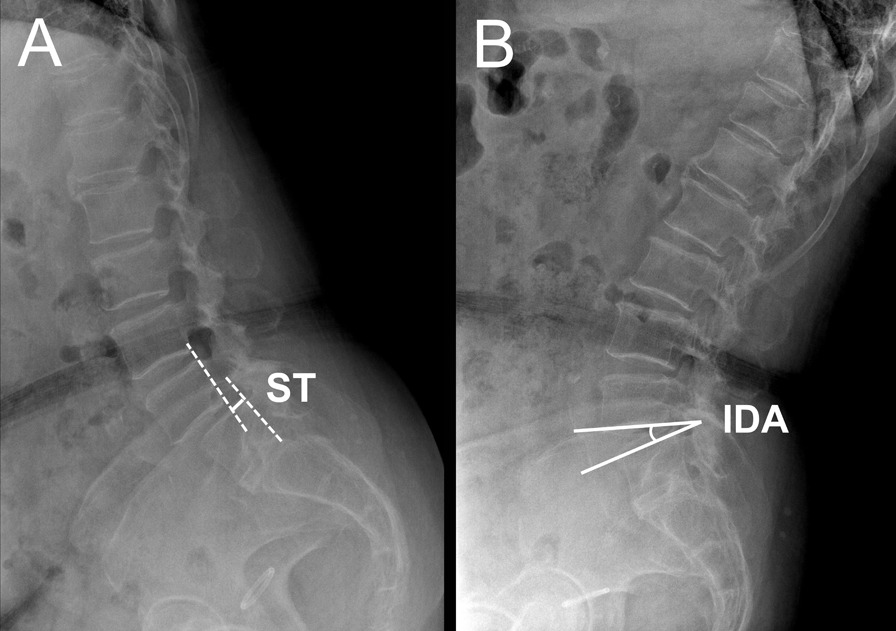
Evaluation of inherent stability of L4/5 segment. **A** ST, sagittal translation. **B** IDA, intervertebral disc angle

The inherent stability of the L4/5 segment was evaluated by flexion–extension radiographs (Fig. 3). The intervertebral disc angle (IDA) indicated the Cobb angle between the inferior endplate of L4 and the superior endplate of L5. Sagittal translation (ST) indicated the interval between two extended lines of the posterior aspect of L4 and L5. Unstable spondylolisthesis was defined as IDA change > 10° or ST change > 3 mm during the flexion–extension of the lumbar spine [13, 20].

All radiographic data measurements were performed by two trained spinal surgeons (Han and Kong), and the average of two measurements was taken as the final result.

### Patient-reported outcome evaluation

The ODI, Japanese Orthopedic Association (JOA) score, VAS-back pain, and VAS-leg pain were examined for all patients at the time of admission by the same experienced research assistant. The validated ODI is a self-administered questionnaire for evaluating back-specific functional disability, consisting of 10 items with scores from 0 to 5 [21]. The value of ODI is less than 20% in the asymptomatic population [22]. The JOA score is a comprehensive assessment method that comprises the rating of subjective symptoms (0–9 scores), clinical signs (0–6 scores), restriction of activities of daily living (0–14 scores), and urinary bladder function (− 6–0 scores) [23]. The JOA score is 29 in ordinary people [23]. The VAS was used to measure back and leg pain for patients based on a 100 mm line, with “painless” (0) and “most severe pain” (100 mm) at each respective end [24].

### Statistical analysis

All data are presented as the mean values ± standard deviation. Normality was tested by the Shapiro‒Wilk Normality test (Additional file 1, Table S1). Univariate and multivariate linear regression analyses were conducted to identify factors relevant to PROs. Categorical variables were represented as sets of dummy variables. Variables with *P* < 0.05 on univariate analysis were included in the subsequent multivariate linear regression analysis. Continuous variables were compared among subgroups using one-way ANOVA and the Kruskal‒Wallis test with Bonferroni or Tamhanes T2 post hoc analysis. The Chi-square test was used to compare categorical variables among groups. Intraclass coefficients and their 95% confidence intervals for all parameters were calculated to evaluate the reliability of the intrinsic variability of radiographic measurements. Values of ICC less than 0.5, between 0.5 and 0.75, between 0.75 and 0.9, and greater than 0.90 are indicative of poor, moderate, good, and excellent reliability, respectively.

Data were analyzed using SPSS Statistics software (version 26.0, IBM Corp., Armonk, NY, USA). Statistical significance was set at a level of *P* < 0.05.

## Results

### Baseline characteristics

In total, 80 (79.2%) females and 21 (20.8%) males were enrolled in the present study, with a mean age of 66.34 ± 8.33 years. All baseline values of the whole cohort are summarized in Table 1. Forty-two of 101 (41.6%) patients were characterized by stable spondylolisthesis, while 59 (58.4%) patients presented unstable spondylolisthesis. Moreover, 49, 38, and 14 patients were classified as coronal balance, LCI, and GCI states, respectively. The vast majority of patients (83.2%) showed a spondylolisthesis of Meyerding grade I, whereas only 16.8% of patients exhibited SP over 25%. All measurements presented high reproducibility, as the values of ICC > 0.85. For the whole cohort, the mean values of ODI and JOA score were 57.84% and 15.96, respectively. The VAS scores of back pain and leg pain reached approximately 62 mm and 65 mm, respectively.

**Table 1:** Baseline characteristics of patients enrolled in the present study

	Value
Demographic parameters	
Age (years)	66.34 ± 8.33
Female [n (%)]	80 (79.2%)
Male [n (%)]	21 (20.8%)
Height (mm)	160.14 ± 7.54
Weight (kg)	67.16 ± 11.58
BMI (kg/m^2^)	26.16 ± 3.98
Spinal sagittal parameters	
T1S (°)	22.84 ± 6.36
TK (°)	− 35.38 ± 11.28
LL (°)	42.65 ± 12.78
SS (°)	30.07 ± 7.72
PT (°)	22.69 ± 8.48
PI (°)	52.39 ± 8.01
PT / PI (%)	44.09 ± 13.80
SVA (mm)	26.86 ± 41.97
SLL (°)	14.05 ± 6.73
SP (%)	19.40 ± 5.83
Inherent stability	
Stable [n (%)]	42 (41.6%)
Unstable [n (%)]	59 (58.4%)
Spinal coronal alignment	
Balance [n (%)]	49 (48.5%)
LCI [n (%)]	38 (37.6%)
GCI [n (%)]	14 (13.9%)
Meyerding grade	
Grade I [n (%)]	84 (83.2%)
Grade II [n (%)]	17 (16.8%)
Clinical parameters	
ODI (%)	57.84 ± 6.48
JOA score	15.96 ± 1.55
VAS-back pain (mm)	61.56 ± 5.39
VAS-leg pain (mm)	64.96 ± 6.95

*BMI* body mass index, *T1S* T1 slope, *TK* thoracic kyphosis, *LL* lumbar lordosis, *SS* sacral slope, *PT* pelvic tilt, *PI* pelvic incidence, *PT/PI* the ratio of PT to PI, *SVA* sagittal vertical axis, *SLL* segmental lumbar lordosis, *SP* slip percentage, *LCI* local coronal imbalance, *GCI* global coronal imbalance, *ODI* Oswestry disability index, *JOA* Japanese Orthopedic Association, *VAS* visual analogue scale

### Risk factors for worse preoperative PROs

Univariate linear regression analysis was conducted to identify the indicators related to the preoperative PROs. ODI was associated with age, LL, SVA, SLL, and GCI. Age, LL, PT, SVA, SLL, and GCI were related to the JOA score (Table 2). VAS-back pain was correlated with LL, PT, PT/PI, SVA, SLL, unstable spondylolisthesis, LCI, and GCI. For VAS-leg pain, age, LL, SS, PT, PT/PI, SVA, SLL, LCI, and GCI were influential factors (Table 3).

**Table 2:** Univariate linear regression analysis between baseline parameters and ODI / JOA score

	ODI (n = 101)		JOA score (n = 101)	
	B (95% CI)	*P*	B (95% CI)	*P*
Demographic parameters				
Age	0.342 (0.203 to 0.482)	0.000**	− 0.039 (− 0.076 to − 0.003)	0.033*
Male	− 2.359 (− 5.551 to 0.834)	0.146	− 0.013 (− 0.784 to 0.758)	0.973
Height	− 0.090 (− 0.260 to 0.081)	0.147	0.008 (− 0.033 to 0.049)	0.692
Weight	− 0.120 (− 0.229 to − 0.011)	0.052	0.006 (− 0.020 to 0.033)	0.639
BMI	− 0.277 (− 0.597 to 0.043)	0.089	0.007 (− 0.071 to 0.084)	0.865
Spinal sagittal parameters				
T1S	0.175 (− 0.026 to 0.375)	0.087	0.008 (− 0.041 to 0.056)	0.757
TK	0.108 (− 0.005 to 0.220)	0.060	− 0.008 (− 0.035 to 0.020)	0.581
LL	− 0.219 (− 0.310 to − 0.128)	0.000**	0.030 (0.006 to 0.053)	0.013*
SS	− 0.110 (− 0.276 to 0.056)	0.193	0.032 (− 0.007 to 0.072)	0.106
PT	0.126 (− 0.024 to 0.277)	0.099	− 0.037 (− 0.073 to − 0.002)	0.040*
PI	0.035 (− 0.126 to 0.197)	0.663	− 0.014 (− 0.053 to 0.024)	0.458
PT / PI	0.085 (− 0.009 to 0.179)	0.075	− 0.190 (− 0.042 to 0.003)	0.083
SVA	0.103 (0.078 to 0.127)	0.000**	− 0.011 (− 0.018 to − 0.004)	0.002**
SLL	− 0.316 (− 0.498 to − 0.135)	0.001**	0.065 (0.020 to 0.109)	0.005**
SP	− 0.046 (− 0.267 to 0.176)	0.682	− 0.002 (− 0.055 to 0.051)	0.947
Inherent stability				
Unstable	− 0.842 (− 3.445 to 1.762)	0.523	0.054 (− 0.569 to 0.678)	0.863
Spinal coronal alignment				
LCI	1.118 (− 1.486 to 3.722)	0.396	− 0.221 (− 0.824 to 0.381)	0.468
GCI	7.306 (3.655 to 10.957)	0.003**	− 2.041 (− 2.885 to − 1.196)	0.000**

*ODI* Oswestry disability index, *JOA* Japanese Orthopedic Association, *BMI* body mass index, *T1S* T1 slope, *TK* thoracic kyphosis, *LL* lumbar lordosis, *SS* sacral slope, *PT* pelvic tilt, *PI* pelvic incidence, *PT/PI* the ratio of PT to PI, *SVA* sagittal vertical axis, *SLL* segmental lumbar lordosis, *SP* slip percentage, *LCI* local coronal imbalance, *GCI* global coronal imbalance

*P < 0.05

**P < 0.01

**Table 3:** Univariate linear regression analysis between baseline parameters and VAS-back pain / VAS-leg pain

	VAS-back pain (n = 101)		VAS-leg pain (n = 101)	
	B (95% CI)	*P*	B (95% CI)	*P*
Demographic parameters				
Age	0.127 (0.001 to 0.254)	0.049	0.256 (0.098 to 0.414)	0.002**
Male	− 0.579 (− 3.260 to 2.102)	0.669	− 1.135 (− 4.590 to 2.319)	0.516
Height	0.032 (− 0.110 to 0.175)	0.653	− 0.096 (− 0.279 to 0.087)	0.302
Weight	− 0.024 (− 0.117 to 0.068)	0.604	− 0.050 (− 0.169 to 0.070)	0.411
BMI	− 0.115 (− 384 to 0.154)	0.398	− 0.052 (− 0.400 to 0.296)	0.766
Spinal sagittal parameters				
T1S	− 0.117 (− 0.284 to 0.051)	0.170	− 0.097 (− 0.315 to 0.120)	0.377
TK	0.051 (− 0.044 to 0.145)	0.290	0.047 (− 0.075 to 0.170)	0.444
LL	− 0.108 (− 0.189 to -0.026)	0.010*	− 0.207 (− 0.307 to -0.106)	0.000**
SS	− 0.123 (− 0.260 to 0.014)	0.079	(− 0.234 (− 0.408 to (− 0.061)	0.009**
PT	0.201 (0.081 to 0.322)	0.001**	0.209 (0.051 to 0.367)	0.010*
PI	0.106 (− 0.027 to 0.238)	0.116	0.010 (− 0.163 to 0.183)	0.909
PT / PI	0.112 (0.037 to 0.186)	0.004**	0.161 (0.066 to 0.256)	0.001**
SVA	0.031 (0.006 to 0.056)	0.015*	0.037 (0.005 to 0.070)	0.023*
SLL	− 0.201 (− 0.355 to − 0.046)	0.011*	− 0.241 (− 0.441 to (− 0.041)	0.019*
SP	0.064 (− 0.120 to 0.248)	0.490	0.048 (− 0.190 to 0.285)	0.692
Inherent stability				
Unstable	4.716 (2.760 to 6.671)	0.000**	1.114 (− 1.676 to 3.905)	0.430
Spinal coronal alignment				
LCI	2.438 (0.257 to 4.620)	0.029*	6.316 (3.816 to 8.817)	0.000**
GCI	5.551 (2.492 to 8.610)	0.000**	10.102 (6.597 to 13.607)	0.000**

*VAS* visual analogue scale, *BMI* body mass index, *T1S* T1 slope, *TK* thoracic kyphosis, *LL* lumbar lordosis, *SS* sacral slope, *PT* pelvic tilt, *PI* pelvic incidence, *PT/PI* the ratio of PT to PI, *SVA* sagittal vertical axis, *SLL* segmental lumbar lordosis, *SP* slip percentage, *LCI* local coronal imbalance, *GCI* global coronal imbalance

The results of the multiple linear regression are presented in Table 4. Increasing age (B 0.173; 95% CI [0.053 to 0.293]; *P* = 0.005), higher SVA (B 0.084; 95% CI [0.055 to 0.114]; *P* < 0.001), and GCI (B 3.610; 95% CI [0.519 to 6.702]; *P* = 0.023) were independent risk factors for superior preoperative ODI. Patients with GCI (B − 1.703; 95% CI [− 2.686 to − 0.720]; *P* = 0.001) had lower preoperative JOA scores than those with balanced coronal alignment. In terms of preoperative VAS-back pain, unstable spondylolisthesis (B 4.582; 95% CI [2.758 to 6.406]; *P* < 0.001) and GCI (B 4.108; 95% CI [1.032 to 7.185]; *P* = 0.009) were two vital predictors. Additionally, increasing age (B 0.160; 95% CI [0.015 to 0.305]; *P* = 0.031), LCI (B 6.065; 95% CI [3.502 to 8.629]; *P* < 0.001), and GCI (B 8.477; 95% CI [4.608 to 12.346]; *P* < 0.001) were associated with higher preoperative VAS-leg pain.

**Table 4:** Independent influence factors of preoperative patient-reported outcomes in patients with DLS

	B (95% CI)	SE	Beta	t	*P*
ODI^a^					
Age	0.173 (0.053 to 0.293)	0.060	0.222	2.866	0.005**
SVA	0.084 (0.055 to 0.114)	0.015	0.530	5.715	0.000**
Combined with GCI	3.610 (0.519 to 6.702)	1.557	0.193	2.319	0.023*
JOA score^b^					
Combined with GCI	− 1.703 (− 2.686 to − 0.720)	0.495	− 0.382	− 3.439	0.001**
VAS-back pain^c^					
Unstable spondylolisthesis	4.582 (2.758 to 6.406)	0.918	0.421	4.988	0.000**
Combined with GCI	4.108 (1.032 to 7.185)	1.549	0.265	2.652	0.009**
VAS-leg pain^d^					
Age	0.160 (0.015 to 0.305)	0.073	0.191	2.185	0.031*
Combined with LCI	6.065 (3.502 to 8.629)	1.291	0.425	4.698	0.000**
Combined with GCI	8.477 (4.608 to 12.346)	1.948	0.423	4.352	0.000**

*ODI* Oswestry disability index, *SVA* sagittal vertical axis, *GCI* global coronal imbalance, *JOA* Japanese Orthopedic Association, *VAS* visual analogue scale, *LCI* local coronal imbalance

^a^In terms of ODI, increasing age and higher SVA were associated with superior preoperative ODI. Patients with GCI had superior preoperative ODI than those with balanced coronal alignment

^b^In terms of JOA score, patients with GCI had lower preoperative JOA scores than those with balanced coronal alignment

^c^In terms of VAS-back pain, unstable spondylolisthesis was associated with superior preoperative VAS-back pain. Patients with GCI had superior preoperative VAS-back pain scores than those with balanced coronal alignment

^d^In terms of VAS-leg pain, increasing age were associated with superior preoperative VAS-leg pain scores. Patients with LCI or GCI had superior preoperative VAS-leg pain scores than those with balanced coronal alignment

*indicates that *P* < 0.05

**indicates that *P* < 0.01

### Difference analysis among patients with various coronal alignments

**Fig. 4:**
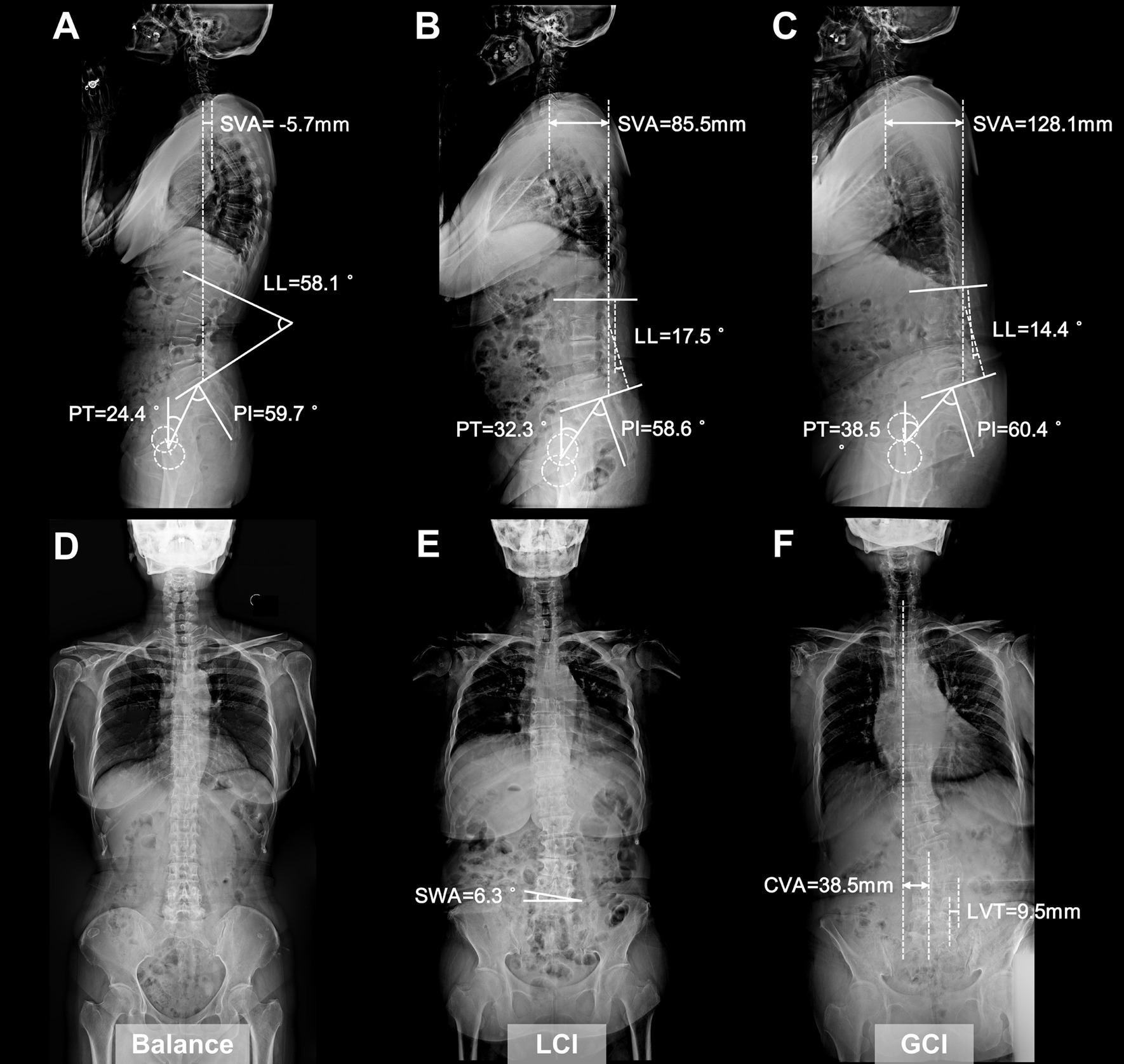
Representative cases with various spinal coronal alignments. **A**, **D** A 65-year-old female with balanced coronal alignment. ODI: 46%, JOA score: 19, VAS-back pain: 60 mm, VAS-leg pain: 55 mm. **B**, **E** A 67-year-old female combined with LCI. ODI: 62%, JOA score: 15, VAS-back pain: 60 mm, VAS-leg pain: 65 mm. **C**, **F** A 71-year-old female combined with GCI. ODI: 65%, JOA score: 14, VAS-back pain: 63 mm, VAS-leg pain: 70 mm

To better understand the characteristics of patients with different coronal alignments, we compared the demographic, radiographic, and clinical parameters among groups (Table 5). Demographic parameters, PI, and the constituent ratio of unstable spondylolisthesis were approximate in all three groups. The GCI group exhibited the highest SVA (*P* = 0.003) with the lowest LL and SLL (*P* = 0.001, 0.002, respectively). In addition, PT and PT/PI increased, while SS decreased significantly (*P* < 0.001) from the balance group to the GCI group, although there was no significant difference between the LCI and GCI groups based on post hoc tests. Furthermore, outcomes of clinical assessment were distinctly worse in the GCI group than in the other groups, including the highest ODI (*P* = 0.005), VAS-back pain (*P* < 0.001), and VAS-leg pain (*P* < 0.001), as well as the lowest JOA score (*P* = 0.006). Representative cases of the three different groups are illustrated in Fig. 4.

**Table 5:** Comparison of demographic, radiological, and clinical parameters among patients with various spinal coronal alignment

	Balance group (n = 49)	LCI group (n = 38)	GCI group (n = 14)	*P*
Demographic parameters				
Age (years)	65.51 ± 7.37	65.74 ± 9.01	70.86 ± 8.75	0.090
Female / Male	37 / 12	32 / 6	12 / 2	0.549
Height (mm)	159.88 ± 7.03	161.11 ± 7.46	158.43 ± 9.49	0.500
Weight (kg)	66.02 ± 11.76	68.99 ± 11.91	66.18 ± 10.06	0.472
BMI (kg/m^2^)	25.85 ± 4.60	26.46 ± 3.33	26.39 ± 3.41	0.762
Spinal sagittal parameters				
T1S (°)	24.04 ± 6.50	22.33 ± 5.00	20.08 ± 8.33	0.100
TK (°)	− 35.20 ± 11.03	− 37.65 ± 9.77	− 29.86 ± 14.52	0.085
LL (°)	46.38 ± 11.89^C^	41.57 ± 10.69	32.39 ± 15.54^A^	0.001**
SS (°)	32.37 ± 7.56^BC^	28.74 ± 7.68^A^	25.61 ± 5.70^A^	0.005**
PT (°)	19.72 ± 7.53^C^	23.39 ± 7.51^C^	31.19 ± 8.39^AB^	0.000**
PI (°)	51.89 ± 7.97	51.44 ± 7.84	56.68 ± 7.79	0.093
PT / PI (%)	37.81 ± 12.44^BC^	48.33 ± 12.65^A^	54.57 ± 10.84^A^	0.000**
SVA (mm)	25.68 ± 35.25^C^	16.04 ± 37.62^C^	60.36 ± 58.13^AB^	0.003**
SLL (°)	15.63 ± 4.99^C^	14.02 ± 6.53^C^	8.56 ± 9.67^AB^	0.002**
SP (°)	19.36 ± 5.31	19.56 ± 6.63	19.13 ± 5.66	0.971
Inherent stability				
Stable / Unstable (n)	21 / 28	15 / 23	6 / 8	0.961
Clinical parameters				
ODI (%)	56.40 ± 6.72^C^	57.52 ± 5.52^C^	63.71 ± 4.94^AB^	0.005**
JOA score	16.33 ± 1.48^C^	16.11 ± 1.43^C^	14.29 ± 0.99^AB^	0.006**
VAS-back pain (mm)	59.88 ± 5.59^C^	62.32 ± 4.59	65.43 ± 4.45^A^	0.000**
VAS-leg pain (mm)	61.18 ± 6.98^BC^	67.50 ± 4.39^AC^	71.29 ± 4.61^AB^	0.000**

*BMI* body mass index, *T1S* T1 slope, *TK* thoracic kyphosis, *LL* lumbar lordosis, *SS* sacral slope, *PT* pelvic tilt, *PI* pelvic incidence, *PT/PI* the ratio of PT to PI, *SVA* sagittal vertical axis, *SLL* segmental lumbar lordosis, *SP* slip percentage, *ODI* Oswestry disability index, *JOA* Japanese Orthopedic Association, *VAS* visual analogue scale

^A^Significant difference compared to Balance group

^B^Significant difference compared to LCI group

^C^Significant difference compared to GCI group

*P < 0.05

**P < 0.01

## Discussion

Preoperative subjective symptoms were accompanied by substantial heterogeneity in patients with DLS, and the relevant contributory factor of worse PROs remains controversial [5, 25]. In this retrospective observational study, the correlation between spinal alignment and preoperative PROs was analyzed in 101 patients with DLS. The results indicated that the PRO-related indicators of ODI, JOA score, VAS-back pain, and VAS-leg pain were not precisely the same. In general, increasing age, higher SVA, unstable spondylolisthesis, and local or global coronal malalignment were associated with worse PRO stratification before surgery.

### Sagittal alignment with preoperative PROs

Spinal sagittal alignment is an essential consideration for the comprehensive assessment of the physiopathology, classification, and treatment options of DLS, but there remains no consensus regarding the relevance between sagittal alignment and clinical symptoms [10, 11]. Multiple sagittal indicators, including LL, SLL, PT, PT/PI, and SVA, were observed to be associated with preoperative PROs in the present study (Tables 2, 3). Similar to the findings of our research, Gille et al. presented that lower LL and higher SVA were associated with worse ODI and short form-12 questionnaire scores in a retrospective study containing 166 patients suffering from DLS [12]. Moreover, SVA was the only sagittal parameter that was an independent influencing factor of preoperative PRO based on our results (Table 4), which highlights the vital implication of global sagittal balance in the evaluation and therapy of DLS. As suggested by previous studies, the sagittal imbalance subtype had the most severe symptoms, and the correction of sagittal deformity to reach a favorable global sagittal alignment was the primary surgical target of such patients [11, 12]. However, in another prospective cohort study of 320 patients diagnosed with DLS, Karim et al. proposed that sagittal malalignment, such as SVA > 50 mm or PI-LL > 10°, did not appear to be correlated with baseline numeric rating scale (NRS) back pain, NRS-leg pain, or ODI. This result might be due to the fact that up to 77% of patients in their cohort had a major complaint of neurogenic claudication with no persistent pain. Thus, more comprehensive studies with larger cohorts are still needed to further elucidate how sagittal alignment affects PROs, although the correlation between higher SVA and worse PROs has been presented in this study.

### Coronal alignment with preoperative PROs

If the degeneration of the intervertebral disc and incompetence of the facet joints evolve asymmetrically, the odds of a combination of coronal malalignment will increase significantly in patients with DLS [26]. Approximately 50% (52/101) of patients showed LCI, and 13.9% (14/101) of patients were simultaneously accompanied by GCI based on our results (Table 1). In contrast, 31.7% and 19.8% of patients had LCI in the retrospective studies of Takahashi et al. and Pan et al., respectively [14, 18]. Higher age was a potential reason for this disparity, as patients with LCI in our cohort were 5–10 years older than patients in the research mentioned above. Moreover, patients with LCI or GCI presented a prominent loss of LL, significant increases in PT/PI and SVA, and worse PROs compared with patients with balanced coronal alignment (Table 5), which was consistent with previous results [14, 18]. Therefore, although LCI and GCI were found to be independent influencing factors of most PROs according to the results of multiple linear regression analysis (Table 4), it cannot be ignored that patients with coronal imbalance were usually accompanied by severe sagittal malalignment and a significant increase in energy expenditure for pelvic compensation; this may have contributed to a higher severity stratification of preoperative symptoms.

### Segmental instability with preoperative PROs

The presence of segmental instability is also common in patients with DLS. As reported by Kirkaldy-Willis, the accumulation of microscopic degenerative damage caused decreased intervertebral disc height and subluxation of the facet joints, which finally led to increased intervertebral instability [27]. Compared with the ratios ranging from 31 to 40% reported by previous studies [28–30], the proportion of unstable spondylolisthesis reached 58.4% in our study (Table 1). These discrepancies might be related to age and racial/ethnic composition. Another issue that merits concern is that the abnormal movement of the diseased segment might contribute to back pain in patients with DLS [31]. In a prospective study of 880 patients with low back pain, Kanemura et al. found that patients with dynamic instability at the L4/5 level exhibited more severe pain than those with stable segments [32]. Moreover, ST change > 3 mm was reported to have the most substantial effect on symptoms, followed by IDA change > 10° [32]. Our observations agree with the previous conclusion that unstable spondylolisthesis is an independent risk factor for higher preoperative VAS-back pain (Table 4). Consistently, Simmonds et al. demonstrated that the degree of back pain aggravated with dynamic stability deteriorated in a qualitative systematic review, and they suggested that decompression and 360° fusion surgery were necessary for patients with unstable DLS [13].

### Age with preoperative PROs

Last but not least, we observed that increasing age was closely associated with higher ODI (Table 4). The patients with GCI were also older than the other groups, although the difference was not significant (Table 5). As previously demonstrated, DLS is the consequence of long-term intersegmental instability at the lumbar motion segment and its predominance in patients older than 50 years [26]. Indeed, the increase in the degree of spinal degeneration with age is another important factor leading to the progression of symptom severity. In summary, DLS patients with increasing age, higher SVA, unstable spondylolisthesis, LCI, or GCI were prone to more severe preoperative subjective symptoms.

There still exist some limitations in our study. First, this was a retrospective study with potential bias. All clinical parameters were evaluated by the same professional research assistant on the patient’s admission. Standardization in the radiographic parameter acquisition was attempted by conducting comprehensive training before the study and illustrating each measurement on the statistical tables. Second, data discussed in the present study were concentrated on patients who were treated by surgical procedure, and there might have been a limited reference value for patients who elected to have conservative treatment. Third, further investigation is still needed to ascertain whether postoperative radiological and clinical outcomes are affected by various preoperative spinal alignments among patients with DLS, and research in this direction is ongoing.

## Conclusion

Among sagittal parameters, SVA was observed as the only independent influencing factor of preoperative PROs in patients with DLS. The absence of segmental stability and the combination of coronal malalignment were associated with worse PROs. Advanced age could also be conducive to the deterioration of preoperative symptoms. In conclusion, DLS patients with higher SVA, unstable spondylolistheses, a combination of LCI/GCI, or increasing age were predisposed to more severe stratification of symptoms before surgery, although marginal differences still existed in regard to specific clinical parameters.

## Supplementary Information

**Additional file 1.** Table S1. Test of normality based on the Shapiro-Wilk approach.

## Data Availability

The datasets used and/or analyzed during the current study available from the corresponding author on reasonable request.

## References

[1] Wiltse LL (1962). The etiology of spondylolisthesis. J Bone Joint Surg Am.

[2] Wang YXJ, Káplár Z, Deng M, Leung JCS (2017). Lumbar degenerative spondylolisthesis epidemiology: a systematic review with a focus on gender-specific and age-specific prevalence. J Orthop Translat.

[3] Rosenberg NJ (1975). Degenerative spondylolisthesis. Predisposing factors. J Bone Joint Surg Am.

[4] Herkowitz HN (1995). Spine update. Degenerative lumbar spondylolisthesis. Spine.

[5] Bydon M, Alvi MA, Goyal A (2019). Degenerative lumbar spondylolisthesis: definition, natural history, conservative management, and surgical treatment. Neurosurg Clin N Am.

[6] Meyerding HW (1933). Diagnosis and roentgenologic evidence in spondylolisthesis. Radiology.

[7] Matsunaga S, Sakou T, Morizono Y, Masuda A, Demirtas AM (1990). Natural history of degenerative spondylolisthesis. Pathogenesis and natural course of the slippage. Spine.

[8] Zhou QS, Sun X, Chen X, et al. How does sagittal spinopelvic alignment of lumbar multisegmental spondylolysis differ from monosegmental spondylolysis? J Neurosurg Spine. 2020:1–8. 10.3171/2020.2.SPINE191415 32302981

[9] Radovanovic I, Urquhart JC, Ganapathy V (2017). Influence of postoperative sagittal balance and spinopelvic parameters on the outcome of patients surgically treated for degenerative lumbar spondylolisthesis. J Neurosurg Spine.

[10] Ferrero E, Ould-Slimane M, Gille O, Guigui P (2015). Sagittal spinopelvic alignment in 654 degenerative spondylolisthesis. Eur Spine J.

[11] Gille O, Challier V, Parent H (2014). Degenerative lumbar spondylolisthesis: cohort of 670 patients, and proposal of a new classification. Orthop Traumatol Surg Res.

[12] Gille O, Bouloussa H, Mazas S (2017). A new classification system for degenerative spondylolisthesis of the lumbar spine. Eur Spine J.

[13] Simmonds AM, Rampersaud YR, Dvorak MF, Dea N, Melnyk AD, Fisher CG (2015). Defining the inherent stability of degenerative spondylolisthesis: a systematic review. J Neurosurg Spine.

[14] Pan W, Zhao JL, Xu J, et al. Lumbar alignment and patient-reported outcomes after single-level transforaminal lumbar interbody fusion for degenerative lumbar spondylolisthesis with and without local coronal imbalance. J Neurosurg Spine. 2020:1–7. 10.3171/2020.7.SPINE20703 33276333

[15] Pearson A, Blood E, Lurie J (2011). Predominant leg pain is associated with better surgical outcomes in degenerative spondylolisthesis and spinal stenosis: results from the Spine Patient Outcomes Research Trial (SPORT). Spine.

[16] Kleinstück FS, Grob D, Lattig F (2009). The influence of preoperative back pain on the outcome of lumbar decompression surgery. Spine.

[17] Kwon O, Lee S, Park SM, Yeom JS, Kim HJ (2022). A complement type to SRS-Schwab adult spinal deformity classification: the failure of pelvic compensation. Spine.

[18] Takahashi T, Hanakita J, Watanabe M, Kawaoka T, Takebe N, Kitahara T (2014). Lumbar alignment and clinical outcome after single level asymmetrical transforaminal lumbar interbody fusion for degenerative spondylolisthesis with local coronal imbalance. Neurol Med Chir (Tokyo).

[19] Zuckerman SL, Cerpa M, Lai CS, Lenke LG (2022). Coronal alignment in adult spinal deformity surgery: definitions, measurements, treatment algorithms, and impact on clinical outcomes. Clin Spine Surg.

[20] Elmose SF, Andersen GO, Carreon LY, Sigmundsson FG, Andersen MO. Radiological definitions of sagittal plane segmental instability in the degenerative lumbar spine—a systematic review. Global Spine J. 2022:21925682221099854. 10.1177/21925682221099854 PMC9972266 35606897

[21] Fairbank JC, Pynsent PB (2000). The Oswestry Disability Index. Spine.

[22] Tonosu J, Takeshita K, Hara N (2012). The normative score and the cut-off value of the Oswestry Disability Index (ODI). Eur Spine J.

[23] Izumida S (1986). Assessment of treatment for low back pain. J Jpn Orthop Assoc..

[24] Jensen MP, Chen C, Brugger AM (2003). Interpretation of visual analog scale ratings and change scores: a reanalysis of two clinical trials of postoperative pain. J Pain.

[25] Karim SM, Fisher C, Glennie A (2022). Preoperative patient-reported outcomes are not associated with sagittal and spinopelvic alignment in degenerative lumbar spondylolisthesis. Spine.

[26] Sengupta DK, Herkowitz HN (2005). Degenerative spondylolisthesis: review of current trends and controversies. Spine.

[27] Kirkaldy-Willis WH, Farfan HF (1982). Instability of the lumbar spine. Clin Orthop Relat Res.

[28] Iii WS, Orías AAE, Shifflett GD (2020). Image-based markers predict dynamic instability in lumbar degenerative spondylolisthesis. Neurospine.

[29] Phan KH, Daubs MD, Kupperman AI, Scott TP, Wang JC (2015). Kinematic analysis of diseased and adjacent segments in degenerative lumbar spondylolisthesis. Spine J.

[30] Ha K-Y, Na K-H, Shin J-H, Kim K-W (2008). Comparison of posterolateral fusion with and without additional posterior lumbar interbody fusion for degenerative lumbar spondylolisthesis. Clin Spine Surg.

[31] Panjabi MM (2003). Clinical spinal instability and low back pain. J Electromyogr Kinesiol.

[32] Kanemura A, Doita M, Kasahara K, Sumi M, Kurosaka M, Iguchi T (2009). The influence of sagittal instability factors on clinical lumbar spinal symptoms. J Spinal Disord Tech.

