# Supplementary material for: The effect of sagittal alignment, coronal balance, and segmental stability on preoperative patient-reported outcomes in patients with degenerative lumbar spondylolisthesis
Source: BMC Surg. 2023 Mar 7;23:48. doi: 10.1186/s12893-023-01947-2 (PMC9990298; doi:10.1186/s12893-023-01947-2)
Supplement: Supplementary file 1 — Additional file 1. Table S1. Test of normality based on the Shapiro-Wilk approach. [file 12893_2023_1947_MOESM1_ESM.docx]

Table S1. Test of normality based on the Shapiro-Wilk approach

|  | Grouping* | Statistic | df | Sig. |
| --- | --- | --- | --- | --- |
| Age | 1 | 0.978 | 49 | 0.497 |
|  | 2 | 0.944 | 38 | 0.055 |
|  | 3 | 0.964 | 14 | 0.794 |
| Height | 1 | 0.981 | 49 | 0.587 |
|  | 2 | 0.947 | 38 | 0.070 |
|  | 3 | 0.824 | 14 | 0.010 |
| Weight | 1 | 0.975 | 49 | 0.367 |
|  | 2 | 0.958 | 38 | 0.159 |
|  | 3 | 0.942 | 14 | 0.441 |
| BMI | 1 | 0.953 | 49 | 0.051 |
|  | 2 | 0.975 | 38 | 0.532 |
|  | 3 | 0.956 | 14 | 0.662 |
| T1S | 1 | 0.965 | 49 | 0.149 |
|  | 2 | 0.978 | 38 | 0.652 |
|  | 3 | 0.95 | 14 | 0.564 |
| TK | 1 | 0.989 | 49 | 0.935 |
|  | 2 | 0.951 | 38 | 0.093 |
|  | 3 | 0.905 | 14 | 0.135 |
| LL | 1 | 0.974 | 49 | 0.350 |
|  | 2 | 0.984 | 38 | 0.849 |
|  | 3 | 0.939 | 14 | 0.410 |
| SS | 1 | 0.98 | 49 | 0.564 |
|  | 2 | 0.981 | 38 | 0.751 |
|  | 3 | 0.962 | 14 | 0.749 |
| PT | 1 | 0.969 | 49 | 0.215 |
|  | 2 | 0.968 | 38 | 0.340 |
|  | 3 | 0.968 | 14 | 0.849 |
| PI | 1 | 0.983 | 49 | 0.713 |
|  | 2 | 0.957 | 38 | 0.152 |
|  | 3 | 0.926 | 14 | 0.267 |
| SVA | 1 | 0.979 | 49 | 0.510 |
|  | 2 | 0.972 | 38 | 0.455 |
|  | 3 | 0.951 | 14 | 0.573 |
| SLL | 1 | 0.99 | 49 | 0.957 |
|  | 2 | 0.959 | 38 | 0.173 |
|  | 3 | 0.924 | 14 | 0.250 |
| SP | 1 | 0.979 | 49 | 0.520 |
|  | 2 | 0.967 | 38 | 0.310 |
|  | 3 | 0.947 | 14 | 0.522 |
| PT/PI | 1 | 0.983 | 49 | 0.694 |
|  | 2 | 0.973 | 38 | 0.474 |
|  | 3 | 0.889 | 14 | 0.079 |
| ODI | 1 | 0.979 | 49 | 0.526 |
|  | 2 | 0.952 | 38 | 0.100 |
|  | 3 | 0.91 | 14 | 0.157 |
| VAS-Back pain | 1 | 0.873 | 49 | 0.000 |
|  | 2 | 0.927 | 38 | 0.016 |
|  | 3 | 0.9 | 14 | 0.111 |
| VAS-Leg pain | 1 | 0.939 | 49 | 0.013 |
|  | 2 | 0.933 | 38 | 0.026 |
|  | 3 | 0.882 | 14 | 0.063 |
| JOA score | 1 | 0.849 | 49 | 0.000 |
|  | 2 | 0.918 | 38 | 0.009 |
|  | 3 | 0.892 | 14 | 0.087 |

BMI, body mass index; T1S, T1 slope; TK, thoracic kyphosis; LL, lumbar lordosis; SS, sacral slope; PT, pelvic tilt; PI, pelvic incidence; SVA, sagittal vertical axis; SLL, segmental lumbar lordosis; SP, slip percentage; PT/PI, the ratio of PT to PI; ODI, Oswestry disability index; VAS, visual analogue scale; JOA, Japanese Orthopedic Association.

*Group 1 indicated the balance group; Group 2 indicated the local coronal imbalance group; Group 3 indicated the global coronal imbalance group.
